# Management and Prognosis of Patients with Mild Traumatic Brain Injury: A Narrative Review

**DOI:** 10.3390/brainsci16030273

**Published:** 2026-02-28

**Authors:** Mayank Gupta, Sara Khan, Samantha Bunk, Anand Patil, Joan Stilling, Jaspal Singh, Sudhir Diwan, Michael Schatman, Anushka Bajaj, Alaa Abd-Elsayed, Steven Kosa

**Affiliations:** 1 Kansas Pain Management & Neuroscience Research Center, LLC, Overland Park, KS 66215, USA; mayank.g@kansaspainmanagement.com; 2 College of Osteopathic Medicine, Kansas City University, Kansas City, MO 64106, USA; samantha.currier@ohiohealth.com; 3 Department of Physical Medicine and Rehabilitation, St. Luke’s Rehabilitation Medical Center, Spokane, WA 99202, USA; 4 Rehabilitation Medicine, Weill Cornell Medical College, New York, NY 10032, USA; qsi9001@med.cornell.edu (J.S.); jrs9012@med.cornell.edu (J.S.); 5 Advanced Spine on Park Avenue, New York, NY 10461, USA; sudhir.diwan63@gmail.com; 6 Department of Anesthesiology, Perioperative Care & Pain Medicine, New York University School of Medicine, New York, NY 10016, USA; michael.schatman@nyulangone.org; 7 Undergraduate Education, University of California, San Diego, CA 92037, USA; anushkabajaj10@gmail.com; 8 Department of Anesthesia, University of Wisconsin, Madison, WI 53706, USA; alaaawny@hotmail.com; 9 North Kansas City Hospital, North Kansas City, MO 64116, USA; steven.kosa@meritashealth.com

**Keywords:** mild traumatic brain injury, prognostic factors, diagnostic criteria, management

## Abstract

**Highlights:**

**What are the main findings?**

- Despite mild traumatic brain injuries having a set diagnostic criterion, several poor prognostic factors greatly influence recovery timelines.
- These prognostic factors include injury-related factors, patient-related factors, and contextual psychosocial factors.

**What are the implications of the main findings?**

- The poor prognostic factors should be taken into consideration when treating patients with mild traumatic brain injuries.
- Approach to mild traumatic brain injuries should be approached with patient-centered management efforts to manage care adequately.

**Abstract:**

**Background/Objectives**: Mild traumatic brain injury (mTBI) is the most common subtype of traumatic brain injury, where patients experience a multitude of symptoms from headaches to memory loss and mood changes. Consequently, there are known poor prognostic factors for mTBI that can impede recovery and alter management courses. This narrative review aims to synthesize and provide a critical assessment of the current diagnostic criteria, management, and prognostic factors for mTBI to inform practice guidelines. **Methods**: This study adopts a patient-centered approach, focusing on treating presenting symptoms and referring patients to specialists for abnormal exam findings as needed. These findings are based on a narrative review of existing literature and the medical opinions of experts in neurology, physical medicine and rehabilitation, and pain medicine. The evidence supports that there are patient-related, injury-related, and contextual psychosocial factors that further complicate the long-term prognosis and management of mTBI. **Conclusions**: mTBI is defined by a set of diagnostic criteria: post-traumatic amnesia (PTA) lasting no longer than 24 h, loss of consciousness (LOC) not exceeding 30 min when present, and a Glasgow Coma Scale (GCS) score between 13 and 15. Current treatment options include prescribed rest followed by a gradual return to physical activity, medication management for symptoms with cognitive behavioral therapy, or vestibular physical therapy. Notably, several of these diagnostic criteria overlap with known poor prognostic indicators. These prognostic factors can be grouped into three categories: injury-related factors (LOC, positive imaging findings, history of prior concussions, and high symptom burden); patient-related factors (demographic characteristics and psychiatric history); and contextual psychosocial factors.

## 1. Introduction

In the United States, there are 2.5 million traumatic brain injuries (TBIs) every year [1]. Approximately 70 to 90 percent of traumatic brain injuries are diagnosed as mild traumatic brain injuries (mTBI) [2]. The term “concussion” is often interchangeable with the term “mild traumatic brain injury” since concussive injury is considered to be a subset of mTBI due to its self-limiting nature [3]. In the most general sense of the word, a concussion results from brain injury secondary to a common trauma or sports-related injury that occurs across all age groups [3]. Many cases are made for distinguishing concussion from mTBI by emphasizing concussion as transient clinical symptoms with impairments to function, instead of being classified as a structural injury [4,5].

Although mTBIs are classified as “mild,” people experience heterogeneous symptoms that affect cognitive, physical, and neuropsychological functioning [6]. These symptoms can include headaches, fatigue, inattentiveness, memory loss, brain fog, sleep disturbances, and mood dysregulation, with short- and long-term repercussions for one’s quality of life [7]. Diagnosing an mTBI relies heavily on a clinical diagnosis due to imaging failing to detect attributable abnormalities within patients with mTBIs [8]. Treatment and management often involve a multidisciplinary team to address the complex symptoms.

The recent growing media attention on the topic of the adverse and severe consequences of repetitive TBI being linked to neurodegenerative diseases such as Chronic Traumatic Encephalopathy (CTE) has led to further exploration of poor prognostic factors [9]. Even when stratified by severity, mTBIs increase the risk of the development of dementia by up to 4-fold [10,11]. At this time, there is still a large controversy surrounding the direct association between TBI and dementia or CTE, and there is conflicting information in the literature [11]. Studies have suggested that the attributable risk of dementia in patients who were hospitalized for a TBI is up to 15% [11]. There is now a focus in current research on identifying risk factors for which demographic will suffer a poor prognosis from an mTBI.

This paper aims to review the management and prognosis of patients diagnosed with mTBI. Diagnostic criteria, management, and prognostic factors will be examined to better understand who is at risk for a poor prognosis of mTBI and support improved outcomes in this patient population.

## 2. Materials and Methods

A narrative review methodology was selected due to the multidisciplinary nature of the research question. The narrative review allows for integration of diagnostic criteria, management, and poor prognostic factors within a single clinical framework. The heterogeneity of the domains ranges from clinical assessment tools and imaging modalities to serum biomarkers, pharmacologic and non-pharmacologic therapies, and psychosocial influences. Given this wide scope, a formal systematic review with quantitative synthesis would have required narrowing the research question to a single intervention or outcome. The intent of this review was integrative and conceptual rather than to generate pooled effect sizes. Accordingly, a structured narrative approach was used.

**Figure 1:**
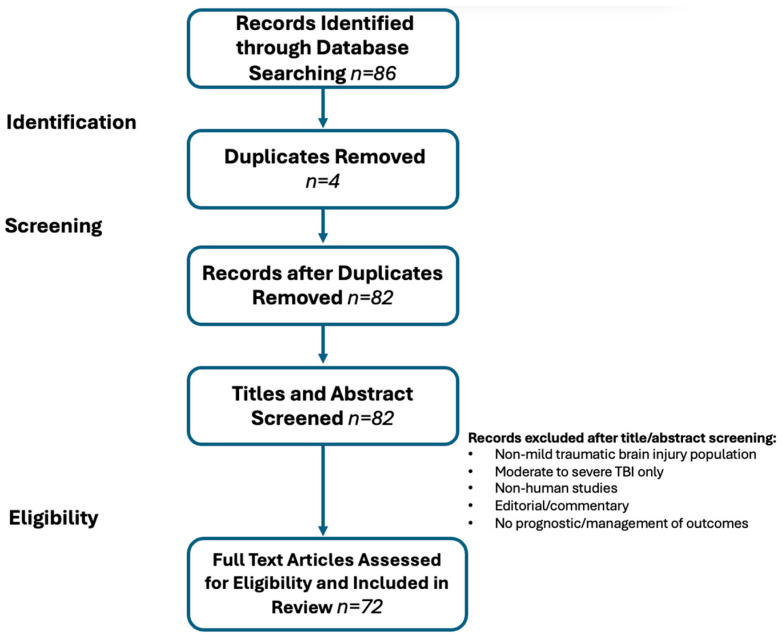
Study Selection Flow Diagram: 86 records were identified through database searching. After removing four duplicates, 82 records were identified through database searches. After removing 4 duplicates, 82 articles underwent title and abstract screening. 10 records were excluded based on the eligibility criteria, and 72 articles were included in the final assessment.

A focused literature search was conducted between 1 April 2025 and 20 February 2026, using PubMed/MEDLINE, Embase, and Google Scholar. Studies were not restricted by publication year; rather, all relevant peer-reviewed literature meeting eligibility criteria was considered, regardless of publication date. Foundational consensus statements and landmark trials were included to ensure a comprehensive historical context. Keywords include “mild traumatic brain injury”, “concussion”, “diagnosis”, “recovery”, “prognostic factors”, and “treatment” were used in various combinations (Supplementary File S1). The exclusion criteria include non-English studies, conference abstracts without full text, and non-human studies. Titles and abstracts were imported into Mendeley and screened for relevance and duplication; eligible studies were thoroughly reviewed (Figure 1).

After identifying 86 records through database searches, 4 duplicates were removed, leaving 82 articles for the final review. The 72 studies included in this narrative synthesis predominantly evaluated general adult civilian populations with mTBI (n = 68). Two studies evaluated a military service member population, and two studies examined a pediatric cohort with sports-related injuries. Although mechanisms of injury were not formally extracted, common mechanisms in the civilian population included falls, motor vehicle collisions, and sports-related injuries. Overall, studies found that falls were more commonly reported in older adult cohorts, whereas sports-related mechanisms were more prevalent in younger populations.

Furthermore, a formal quantitative risk-of-bias assessment tool (e.g., GRADE or Cochrane Risk of Bias) or weight was not applied due to the narrative design and variety of included study designs and outcomes. However, an informal qualitative evidence appraisal framework was used during synthesis, in which studies were prioritized based on methodological rigor and clinical relevance. These included randomized controlled trials, large prospective cohort studies, and systematic reviews. Smaller studies, including case series and exploratory biomarker studies, were not excluded from review. Guideline-based consensus statements and position papers from recognized professional organizations were used to anchor diagnostic and management recommendations. When conflicting findings were identified, preference was given to higher-level evidence and studies with larger sample sizes or more robust methodology.

Given the narrative nature of this review, findings were organized thematically and grouped into major domains of interest: 1. diagnostic criteria, 2. treatment modalities, and 3. prognostic factors. The literature was summarized within each domain, and areas of consensus and divergence were highlighted.

Expert opinion from the group’s authors was then consulted. The author group is gender-balanced and comprises junior, mid-career, and senior researchers. It includes representatives from different disciplines, including neurology, physical medicine and rehabilitation, and pain medicine. All members are from the United States of America.

## 3. Results

### 3.1. Mild Traumatic Brain Injury Criteria

A mTBI is a brain injury due to external mechanical forces that meets specific diagnostic criteria endorsed by the American Congress of Rehabilitation Medicine and adopted by the World Health Organization (WHO) (Table 1) [12,13]. The first criterion is that post-traumatic amnesia (PTA) must not exceed 24 h [4,12,14]. PTA is an important prognostic factor of functionality and is described as a state of confusion, agitation, loss of attention, and memory loss following an injury [15]. There are many tools available to assess PTA. The Westmead Post-Traumatic Amnesia Scale (WPTAS) utilizes picture cards to measure the duration and presence of PTA [16]. Another popular assessment tool is the Galveston Orientation and Amnesia Test (GOAT), which uses a 0–100 scale to determine when a patient returns from a PTA state [17]. A score of 75 or higher over 2 days indicates resolution of PTA [17]. Lastly, an O-log assessment test is similar to the GOAT but focuses on amnestic and disorientation symptoms; two consecutive scores of 25 or higher indicate resolution of PTA [18].

Loss of consciousness (LOC) is another criterion considered in the diagnostic criteria for mTBI [12]. Although LOC is not required to meet the diagnosis of mTBI, the episode must not exceed 30 min when LOC is present [12,14]. This can often be difficult to ascertain since patients will often have regained consciousness during evaluation and require reliable witnesses.

In addition, a Glasgow Coma Scale (GCS) score between 13 and 15 is measured 30 min after the injury [4,6,12,14]. The original use of the GCS was to assess the level of consciousness. The scale contains motor, eye-opening, and verbal components. Today, the GCS is commonly used to classify the severity of TBI [6]. As a screening tool, the GCS fails to consider pathologic mechanisms of neuronal injury [6]. Subjective symptomology varies greatly between those with a GCS of 13 and those with a GCS of 15, potentially leading to missed opportunities for imaging and further screening in those with a higher GCS [6].

As research continues to advance, more methodologies for describing and characterizing mTBI have emerged. Multidimensional phenotyping of concussions has been proposed as a more precise way to categorize types of mTBI [19]. This method of categorization aims to acknowledge the complexity of diagnosis beyond the standard scales currently available. The main challenge is the lack of standardization, as inclusion criteria vary across the literature [19]. Fluid biomarkers have also advanced the ability to accurately determine the severity of brain damage associated with TBI. This technique relies on multiple biomarkers in the patient’s cerebrospinal fluid to help assess the severity of brain damage [20]. Various computational algorithms have been developed to help objectify findings and develop a classification algorithm for mTBI [21]. While advancements in these modalities continue, diagnosis and treatment of mTBI remain largely reliant on the criteria discussed above.

### 3.2. Imaging and Laboratory Evaluation

Imaging is not routinely recommended for diagnosing mTBI, as it is diagnosed clinically using the parameters stated above [8]. Imaging with the use of computed tomography (CT) is only utilized in patients with red flag symptoms to rule out more severe intracranial complications from injury, such as intracranial hemorrhage, contusions, or skull fractures [8]. In the emergency department, the Canadian CT head rule and NEXUS criteria are commonly used to determine who should be evaluated with further imaging for stable trauma [22,23,24]. CT findings are often negative, where approximately 16% of patients with mTBIs will have nonspecific positive findings. These findings vary in severity and can include subdural hematomas, subarachnoid hemorrhages, contusions, epidural hematomas, intraventricular hemorrhages, and petechial hemorrhages [25].

Despite recommendations, imaging of mTBIs can provide insight into the pathologic mechanisms of TBI and provide long-term prognostic value. Magnetic Resonance Imaging (MRI) is more sensitive than CT for detecting microstructural changes [26]. Patients with mTBIs may commonly reveal white matter hyperintensities on MRI [27]. Additionally, susceptibility-weighted imaging can reveal microhemorrhages [26,28]. Diffusion tensor imaging (DTI) is another subtype of MRI modality that shows promising use for assessing white matter integrity in mTBI [29]. However, it is not readily available to most institutions [29]. Therefore, variability in acquisition protocols, lack of standardized normative thresholds, and limited prognostic validation currently restrict the routine clinical use of DTI for individual patient management.

Quantitative EEG (qEEG) can help quantify and track patients’ progress with mTBI [30]. qEEG measures alterations in brain wave patterns, where several patterns have been uniquely recorded in patients with mTBI [30]. These waveforms show increased global theta power and decreased alpha power, along with increased beta-band interhemispheric coherence [30]. Patients with mTBI also exhibit abnormal alpha desynchronization and synchronization during working memory tasks [31]. Although these new imaging techniques are not widely accessible, there is promise for future research integrating them for mTBI. While these findings provide insight into functional disruption, qEEG remains primarily a research tool, and its role in routine clinical practice has not been standardized.

New laboratory findings and biomarkers are being investigated to support mTBI diagnosis further and provide prognostic insights. Among these markers is the S100B subunit, which shows 91 percent sensitivity and has potential for implementation in screening tools [32]. Two additional markers, glial fibrillary acidic protein (GFAP) and ubiquitin C-terminal hydrolase (UCH-L1), have received FDA clearance for use in the acute evaluation of mild head trauma to help identify patients at low risk of intracranial injury who may not require CT imaging [32,33]. Importantly, this clearance applies to acute triage decision-making rather than long-term prognostic assessment. Although elevated biomarker levels may correlate with injury severity, validated thresholds for predicting prolonged symptom duration or functional recovery remain limited.

At present, advanced neuroimaging modalities (DTI, qEEG) and serum biomarkers should be considered adjunctive or investigational in the context of outpatient prognostic evaluation. Clinical assessment and established risk factors remain the cornerstone of mTBI diagnosis and risk stratification pending further validation of these emerging tools.

### 3.3. Management

#### 3.3.1. Prescribed Rest and Return to Physical Activity

**Figure 2:**
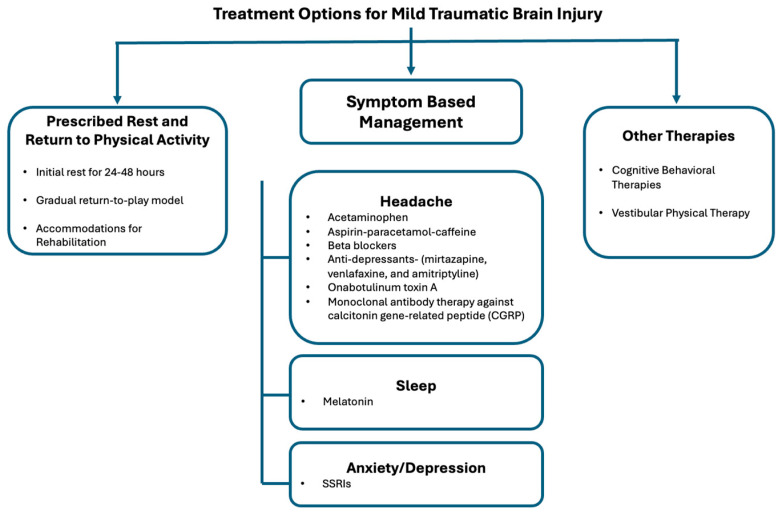
Treatment Options for Mild Traumatic Brain Injury. Management strategies for mTBI may be conceptualized along two pathways: (1) a temporal recovery model consisting of brief relative rest (24–48 h) followed by graded, symptom-threshold return to activity; and (2) symptom-targeted interventions addressing headache, sleep disturbance, and mood disorders of anxiety and depression. Cognitive behavioral therapy and vestibular physical therapy case series and meta-analyses offer non-medication modalities that can further manage post mTBI mood symptoms, fatigue, dizziness, and imbalance.

While the medical opinion on how to treat mTBIs has evolved throughout the past several decades, the recommendation for patients to engage in complete physical and cognitive rest for the first 24 to 48 h following an injury has remained consistent (Figure 2) [8]. This recommendation helps alleviate symptoms by decreasing the brain’s metabolic use [8]. Most importantly, the brain’s chemical homeostasis of glutamate and calcium is disrupted following a traumatic brain injury. As a result, it is more vulnerable to any other traumatic event or added stress [34]. Second impact syndrome is a condition that occurs when a second insult to the brain occurs before the first injury has had time to heal [34]. Athletes returning to their sports too early are at high risk for second impact syndrome [34].

While reducing risk for secondary injury to the brain is essential after a concussion, there is evidence that complete rest is not beneficial [8]. Levels of brain-derived neurotrophic factor (BDNF), a neuron growth and repair promoter, were found to be increased in the weeks following initiation of aerobic exercise, suggesting a potential link between voluntary exercise after mTBI and enhanced brain neuroplasticity [35]. An example of this phenomenon can be found in a 2009 study, in which the Buffalo Concussion Treadmill Test (BCTT) was used to allow one group of participants to complete 20 min of sub-symptomatic aerobic exercise, while another group strictly rested and was reevaluated weekly to determine when they became asymptomatic and could return to activity [35]. The BCTT allowed individuals to exercise at a level that would not cause excessive stress on the brain by monitoring and restricting each subject’s heart rate within a desired range [35]. It was found that individuals who engaged in this limited aerobic exercise recovered faster and demonstrated greater cognitive performance than those who strictly rested [35].

The optimal exercise dose for mTBI involves prescribing aerobic exercise, such as walking or stationary cycling, at 50% of age-predicted maximum heart rate. Dosing intervals should keep activity intensity below a 2-point increase in symptom exacerbation, or what is considered “mild” exacerbation [5]. Exercise should be initiated 24 to 72 h after injury [5]. If the patient has mTBI-related symptoms, exercise should still be encouraged during this time frame, as early light activity is more effective than rest [5]. Many foundational organizations, including the Ontario Neurotrauma Foundation, CDC, and the Sixth International Conference on Concussion in Sport, recommend prescribing physical activity and aerobic exercise to facilitate recovery and reduce the incidence of symptoms persisting for more than 4 weeks [5].

In randomized control trials for adolescents, those prescribed aerobic exercise two to 10 days after a concussion recovered faster than those assigned to stretching [5]. Contrastingly, another randomized trial in adults prescribed 30 min of daily light exercise within 48 h of injury but found no difference in persistent post-concussion symptoms at 30 days. Suggesting that prescribed exercise without individualized heart rate thresholds or formal monitoring may be insufficient [36]. Patient selection is broad and incorporated into a multidisciplinary approach, regardless of initial symptom burden, and works well in patients with persisting symptoms [5].

In light of these studies, extensive cognitive and/or physical rest following a traumatic brain injury is now cautioned against as it can delay recovery [8]. This is important as delayed recovery could lead to poor prognostic factors in mTBI. While initial rest during the acute phase of injury is required, patients should be encouraged to resume activity on a voluntary, self-limited basis [8]. This is also known as a “return-to-play” model [8]. To facilitate this, physicians should assist patients in advocating for academic and work accommodations to create space for cognitive and physical rehabilitation at a pace they can tolerate [8].

#### 3.3.2. Medication Management

Currently, there are no FDA-approved medications for the treatment of mTBI [37]. However, several medications have been utilized for their off-label use in mTBI neuropsychiatric symptoms (Figure 2) [37]. When addressing low-grade inflammation after a mTBI, patients can be prescribed anti-inflammatory and analgesic compounds [8]. Patients with mTBI are susceptible to various headache types [8]. Generally, medications such as acetaminophen are sufficient for pain control [8]. However, patients must be mindful of continued use of these medications since repeated use can lead to medication overuse headaches (MOH), which could worsen pre-existing mTBI symptoms [8].

For more severe headaches in which these over-the-counter medications do not adequately provide analgesia, treatment should focus on the headache subtype the patient reports. Various medications can be used depending on the number of headache days per month and the goal of acute versus preventive treatment. For both tension-like and migraine-like headache subtypes, aspirin-paracetamol-caffeine combination pills are helpful for acute treatment, while amitriptyline can be used for prevention. Other antidepressants, such as mirtazapine and venlafaxine, may also be recommended for tension-like headaches. In addition to amitriptyline, beta blockers are also effective for the prevention of migraine-like headaches. Monoclonal antibody therapy against calcitonin gene-related peptide (CGRP) can also be considered for prophylaxis [38]. Onabotulinum Toxin A is approved by the United States Food and Drug Administration (FDA) to treat chronic migraines and thus could be utilized for managing the migraine-like subtype of headaches experienced by mTBI patients [39].

mTBI patients can use melatonin to relieve sleep disturbances (Figure 2) [40]. Melatonin was found to be safe and effective in improving sleep quality over 4 weeks in patients with TBI [41]. Sleep disturbances were significantly reduced with 2 mg of prolonged-release melatonin taken nightly, 2 h before bedtime [41].

Cognitive and physiological symptoms of anxiety and depression can further exacerbate mTBI symptoms [37]. Consequently, confounding symptoms of loss of attention and brain fog are multifaceted and are also common symptoms of general anxiety and depression [37]. Studies have shown that antidepressants can be utilized to not only address these confounding symptoms but also to compensate for transmitters like serotonin, where signaling pathways may be disrupted or altered following a TBI [37]. Selective serotonin reuptake inhibitors (SSRIs) are commonly prescribed for their role in the treatment of mTBI with depression or anxiety symptoms (Figure 2) [37]. Adjunctive therapy of medication use with cognitive behavioral therapy (CBT) can significantly reduce neuropsychiatric symptoms experienced in mTBI (Figure 2).

#### 3.3.3. Cognitive Behavioral Therapy (CBT)

CBT is emerging as one of the mainstay treatments for people who experience neuropsychological symptoms like anxiety and depression with mTBI [42]. Specifically, CBT can be beneficial in addressing prolonged post-concussive symptoms experienced in mTBI [43]. In chronic cases, the symptoms patients experience are no longer just a result of the structural damage sustained by the injury but rather complicated by accompanying psychological distress [44]. CBT can potentially be used to target negative thought processes and maladaptive coping mechanisms by creating strategies to reframe and manage their mTBI symptoms. Maladaptive beliefs can lead to patients finding themselves in “vicious cycles” contributing to their symptom evolution and consequent avoidance behavior in adhering to mTBI treatment [44]. The purpose of CBT is to address these concerns and systematically challenge these beliefs in order to break this cycle and increase patient morale [44]. One study found that 12 sessions significantly improved fatigue, anxiety, and post-concussive symptoms in patients diagnosed with mild to moderate mTBI [43]. Patients experienced more benefit when the sessions were completed over a shorter period [43]. CBT can be cognitively demanding for patients, as they are required to challenge core beliefs during a vulnerable time. Clinicians must be cognizant of this to prevent additional psychological distress or worsening existing anxiety or depression. In a randomized clinical trial conducted amongst military members experiencing mTBI, it was found that patients in the CBT group had high adherence and low dropout levels similar to the other groups [45]. This is despite their treatment being more extensive and cognitively challenging than other models of therapy and psychoeducation [45]. This indicates that CBT can be an acceptable and manageable form of treatment for mTBI patients with little safety concern [45,46]. One way to protect patients’ health is to conduct feedback sessions after treatment, so patients can share their experience with the clinician to help adjust future treatment techniques [44]. This can be an opportunity for the clinician to ensure sessions are modeled to support the patient’s treatment goals [44].

#### 3.3.4. Vestibular Physical Therapy (VPT)

mTBI can cause neurological changes, especially when the vestibular system is affected. Disrupting the vestibular system can lead to dizziness, imbalance, and changes in gait [47]. For patients experiencing these deficits, VPT is often recommended (Figure 2). VPT facilitates compensation and helps patients re-train their balance through specific exercises and activities [47]. These tasks can be tailored to patients’ deficits and abilities [48]. Similar to CBT, VPT relies on graded exposure principles and protocols designed by clinicians, which are titrated to the patient’s symptom profile [48]. To protect patients’ safety, it is important that the clinician modify exercises based on the patient’s individual symptom tolerance [48]. While temporary deficits such as dizziness or nausea are expected during exercises that challenge the vestibular system, it is important to pace patients appropriately so that symptom provocation occurs in a controlled manner [48]. An example of this is a physical therapist may recommend habituation exercises for a motion-sensitive patient [48]. Habituation exercises allow motion-sensitive patients to practice recalibration of the compromised central nervous system [48]. While some dizziness is expected, patients’ symptoms must be monitored throughout the session to prevent further trauma [48]. VPT is delivered over multiple sessions, and patient adherence depends on progressively increasing their window of tolerance through these exercises. VPT may be paired with light aerobic exercise and positive psychology [47]. Aerobic exercise improves one’s awareness of their body, especially after injury, whereas positive psychology helps improve a patient’s ability to cope with symptoms, especially with the challenges of VPT [48].

### 3.4. Long-Term Prognostic Factors

**Figure 3:**
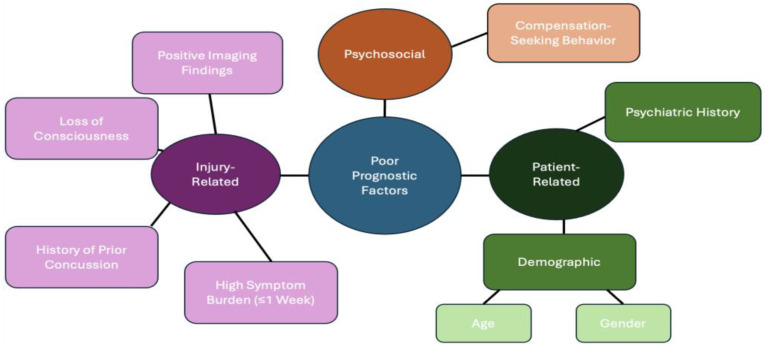
Poor Prognostic Factors for Mild Traumatic Brain Injury. Poor recovery after mTBI is influenced by injury-related, patient-related, and psychosocial factors. Injury-related variables have been strongly defined to include loss of consciousness, positive neuroimaging findings, prior concussions, and high early symptom burden. Patient-related factors include psychiatric history and demographic variables such as age and sex. Psychosocial influences, including compensation-seeking behavior and perceived injustice, may further modulate symptom persistence. These domains interact dynamically rather than independently and should be considered collectively when risk-stratifying patients.

Several prognostic factors increase the risk for poor outcomes in mTBI (Figure 3). Identifying these risk factors early can enable clinicians to intervene promptly and improve outcomes for patients diagnosed with mTBI. These poor prognostic factors can be categorized into three types: injury-related, patient-related, and contextual psychosocial factors. Injury-related factors describe risk factors regarding the inciting brain injury event (Figure 3). Patient-related factors depict demographic and comorbid conditions that can lead to a poor prognosis of mTBI. Contextual psychosocial factors include environmental, social, and psychological influences that can contribute to prolonged symptomology following mTBI.

#### 3.4.1. Injury-Related Factors

##### Loss of Consciousness (LOC)

LOC at the time of injury was a significantly poor prognostic factor of mTBI [49,50]. LOC was shown to have incomplete recovery at one month and three months [51]. Symptoms, such as cognitive and memory deficits, persist with LOC following mTBI [52]. The duration of unconsciousness that individuals experience affects the severity and prognosis [52]. Where LOC greater than 30 min changes the diagnostic criteria from a mTBI to moderate or severe TBI [4]. As previously mentioned, this diagnostic criterion requires a reliable witness and can be challenging to ascertain. The American Academy of Neurology acknowledges that LOC can prolong recovery time and cause long-lasting cognitive deficits and should be considered in poor prognosis with mTBI management [53].

##### Positive Image Findings

Positive CT scans reporting intracranial abnormalities lead to poor prognostic factors for people with mTBI [54]. More specifically, patients with findings of petechial hemorrhage, subarachnoid hemorrhage, subdural hematoma, and even subtle microhemorrhages and contusions had incomplete recovery of up to one year post-concussion and experienced more severe cognitive impairment [55]. mTBI image findings can be subtle and may not be revealed on CT, where 27 percent of mTBI patients with initial negative CT findings at the emergency department had abnormal initial brain MRIs [56]. CT phenotypes can be described based on the biomechanics of the injury. Subarachnoid hemorrhage, contusion, and subdural hematoma primarily occur in patients with linear acceleration and deceleration. These injury biomechanics were linked to incomplete recovery at 6 months and greater degrees of unfavorable outcomes at 2 years or more [57]. On the other hand, intraventricular hemorrhage and petechial hemorrhage are most likely attributed to biomechanical injury from rotational forces [57]. More specifically, intraventricular hemorrhage causes injury to deeper structures, suggesting more severe biomechanical rotation forces [57]. These injury phenotypes were associated with poor outcomes at all time periods up to 12 months [57]. Contrastingly, epidural hemorrhage is shown to have early incomplete recovery, but not with poor prognosis at any point after injury [57]. MRIs that depicted contusions and or greater than four foci of hemorrhagic axonal injury were associated with poor outcomes three months after the inciting event [56].

##### History of Prior Concussion

A recent history of prior concussion not only increases the risk of sustaining a subsequent concussion but is a significant poor prognostic factor for mTBI [58]. In adolescents, it was found that having a concussion within the last year led to a median symptom duration of 35 days compared to those without a history of previous concussion of 12 days [58]. Single mTBIs often have a good prognosis [59]. However, even though the TBI is classified as “mild”, recurrent mTBIs can have a cumulative effect [59]. Recurrent mTBIs are shown to increase cognitive impairment, mood dysregulation, and psychiatric conditions, and increase the long-term incidence of CTE [59]. Awareness of repetitive head injury consequences has resulted in strict regulations for athletes with stringent return-to-play policies to decrease the risk of repetitive injury [59].

##### Symptom Burden

Initial symptom burden at the time of injury and within the first week has been linked to increased risk of prolonged mTBI recovery [59]. Post-concussion scores include cognitive, affective, somatic, and sleep symptoms to calculate symptom burden and are typically used within the first three weeks of a mTBI [60]. A higher score was shown to predict a longer recovery from mTBI, within a 28-day recovery period [60]. Symptoms, including immediate dizziness, migraines, fogginess, vomiting, numbness, and tingling, were associated with slower reaction times, memory impairment, and slower processing speed, with prolonged symptom resolution [60]. Also, high pain intensity at the time of injury has a 6-fold increase in the risk of developing chronic pain from an mTBI [61]. Patients with high-intensity pain reported more mood and behavioral dysregulation symptoms, including aggressiveness, depression, anxiety, and paranoia [61].

#### 3.4.2. Patient-Related Factors

##### Demographic

A. Age

As we age, there is an increased fall risk, with falls being the most common cause of sustaining an mTBI in adults aged 65 and older [62]. Age is considered a predictor of poor outcomes after an mTBI [63]. Poor cognitive recovery persisted for three months in older people at the time of injury [63]. One study found that when comparing MRIs of older patients (mean age 58) with those of younger patients (mean age 26), the older cohort showed distinct patterns of brain activation and poorer working memory performance than the younger cohort [64]. The younger cohort was able to have decreased post-concussive symptoms at follow-up compared to the older cohort [64]. Conversely, sustaining a mTBI in one’s early 20 s can result in lifelong neurodegeneration, causing more structural brain degeneration long-term than those who sustain a mTBI at an older age [65]. Therefore, there are distinct attributable risks to sustaining an mTBI at both older and younger ages.

B. Gender

Sex-specific differences were observed across multiple studies included in this review. Female patients are more at risk of enduring post-concussive symptoms with sustained mTBI compared to men [50]. Women experienced higher symptom burden with worse cognitive and somatic symptoms due to mTBI compared to men [66,67]. Not only were symptoms worse, but women experienced prolonged symptom duration after an mTBI, independent of reporting symptoms of fatigue and sleep disturbance [66]. Post-concussive symptoms were also worse in women categorized in the 35 to 49 year age group compared to younger and older women [66]. These findings suggest that sex-related biological and psychosocial factors may influence recovery trajectory following mTBI and warrant consideration as poor prognostic factors.

C. Genetic Susceptibility

New literature has suggested a link between genetic factors in predicting functional and cognitive outcomes in mTBI [68,69]. Specifically, the Apolipoprotein E (APOE) ε4 allele gene is involved with neuronal repair and has been heavily studied and associated with persistent impairment in people with mTBI who carry the allele [68]. These dysfunctions include functional parameters such as memory, psychomotor speed, and attention, and behaviors such as sleep disturbance and fatigue [68]. Several other genes, such as BDNF and other polymorphisms, have been identified, but the link to severity remains limited due to the diversity of the patient population [68].

D. Neuroinflammatory Cytokines

In the context of genetics, neuroinflammatory markers play a significant role in neuronal recovery. Most of the literature describes these markers within the context of traumatic brain injuries without specifically describing mild traumatic brain injuries. Specifically, TNF-α has been cited as an important neuroinflammatory marker in neuronal-mediated cell death but has not been specifically described for its role in mTBI [69,70]. Inflammatory markers of IL-6 were often elevated within the first 24 h of injury in people with mTBI. Specifically, in sports and military populations, this elevation in the marker was more acute, with early elevations at 8 h and a return to baseline in 48 h [70]. In comparison, the general emergency room population showed elevated IL-6 levels lasting up to 6 months after injury [70].

##### Psychiatric History

Having pre-existing anxiety or depression before mTBI is a significant factor for poor prognostic outcomes [71]. Patients with pre-injury anxiety and depression are more likely to develop post-concussive symptoms and experience greater severity of symptoms [72,73]. However, these symptoms may be an extension of their preexisting anxiety and depression, with continuation and increased severity of perceived preinjury symptoms rather than greater somatic post-concussive symptoms [72]. Furthermore, patients with anxiety and depression before mTBI are more likely to develop post-traumatic stress disorder (PTSD) and major depressive disorder (MDD) [74].

Maladaptive coping mechanisms present a challenge in recovery with prolonged mTBI symptoms [71]. Two types of coping mechanisms negatively impact recovery: avoidance coping and endurance coping [75]. Avoidance coping involves avoiding events related to a stressor [75]. This coping style leads to greater perceived disability and poor prognostic outcomes in mTBI [75]. Endurance coping is described as pushing through activities despite experiencing significant physical or emotional distressing symptoms [75]. This maladaptive style leads to overexertion and ultimately delays recovery [75]. People with maladaptive coping mechanisms and comorbid anxiety and depression before mTBI could potentially benefit from cognitive behavioral therapy.

#### 3.4.3. Contextual Psychosocial Factors

Compensation-seeking behavior with litigation presents potential confounders in the poor prognosis of mTBI. More minor improvements are seen in mTBI symptoms when patients are actively involved in litigation, particularly regarding disability ratings [76]. Another study identified a dose–response relationship where higher compensation increased symptom exaggeration and failing malingering indicators [77]. Malingering can present difficulties with clinical evaluation and management of mTBI. Despite scoring similarly on neurocognitive tests, people who were suspected of malingering reported significantly higher ratings of functional impairment [78]. Furthermore, associations between perceived injustice, return to work at three months after injury, and self-reported symptoms were examined in another study [79]. People who reported perceived injustice, which is the belief of being treated unfairly, reported higher self-reported symptoms [79]. Taking into consideration potential opportunities for malingering is essential when understanding the management of mTBI and can provide opportunities for psychological mediation.

## 4. Discussion

The purpose of this narrative review was to gather existing data on diagnosing, managing, and identifying poor prognostic factors in patients with mTBI. Although the prognostic framework has been previously described in the literature, our aim was to synthesize these concepts within the context of evolving diagnostic technologies, emerging biomarker research, and new rehabilitation management. Addressing these poor-prognostic factors across injury-related, patient-related, and contextual psychosocial domains is essential when considering the management of mTBI. This review provides an updated, clinically oriented synthesis, focusing on a patient-centered approach, directing treatment towards presenting symptoms, and referring the patient to specialists for abnormal exam findings as the mainstays of management. Appreciating these interactions can facilitate earlier identification of high-risk patients and support patient-informed management, rather than the isolation of prognostic variables. Intense counseling and education can also help decrease unnecessary healthcare expenditure and reduce patient exposure to harm.

Diagnostic criteria for mTBI appear standardized and consistent across the literature, with an mTBI defined as a GCS of 13–15, LOC < 30 min, PTA < 24 h, and CT imaging showing no structural changes [12]. However, there is new potential for laboratory, genetic, and neuroinflammatory markers to be implemented in the emergency setting [26,32,33]. Regarding treatment, management is based on the return-to-play model, encouraging gradual engagement in physical activity [8,50]. However, management can also be individualized as directed by symptomology of the mTBI, which hosts a variety of treatment options [39,61,72,74].

In terms of poor prognosis factors, there are varying views in the literature as to whether patient demographics, such as young or old age, can lead to a worse prognosis of mTBI [64,65]. However, there is a consensus in the literature regarding patient-related factors, including genetic factors, biomechanics of the injury, psychiatric history, injury-related factors, and psychosocial factors, in predicting the prognosis of mTBI. Generally, patients without poor prognostic factors, including no history of loss of consciousness, no acute findings on CT head scan, and a normal physical examination, should be expected to have the greatest improvement in subjective symptoms between 2 and 12 weeks after injury [80]. Most likely, patients have reached maximal medical benefit within this time. After 12 weeks, follow-up should be minimized, and consideration should be given to the increased risk-versus-benefit of treatment with medications. Furthermore, heterogeneity of patient referral times can lead to suboptimal outcomes [81]. Given that a mTBI significantly impacts a patient’s quality of life at home and work, continued research and development of the standard of care is of utmost importance.

Future prognostic modeling may benefit from the inclusion of biomechanical metrics, genetic susceptibility markers, and neuroinflammatory signals, in conjunction with established clinical guidelines, to further refine outcome prediction in mTBI [44]. Precision medicine in mTBI aims to move beyond traditional clinical features and integrates advanced analytics, machine learning, and electronic medical records to improve outcomes for individuals with mTBI [44]. These frameworks could integrate biomarkers, neuroimaging, and clinical phenotypes to optimize referral strategies, resource allocation, and clinical management. However, challenges arise with data collection from electronic health records, which are “non-stationary,” raising concerns about the need to constantly update models when relying on prior heuristics [44].

For recovery from mTBI, rapidly advancing technologies can assist providers in decision-making for management. These include incorporating emerging treatment domains that use wearable devices to provide real-time biofeedback for vestibular or ocular symptoms. Wearing the device for the first seven days can allow providers to make decisions about return-to-play models and the progress of rehabilitation [81].

### Limitations

Narrative reviews pose several limitations. First, we did not utilize formal risk-of-bias assessment tools or quantitative meta-analysis. Second, we excluded non-English articles. Restricting the review to English-language articles introduces a language bias and limits global generalizability. Additionally, outdated sources, defined as over 10 years old, can provide confounding or out-of-date recommendations. However, citing sources for original ideas can often date back decades and be strengthened with supportive evidence from current studies. Population cohorts exist within the mTBI literature from sports-related, civilian, and military-related head trauma, which have varying baselines of psychiatric burden and healthcare access. These demographics may influence treatment responsiveness and prognosis. Although the majority of our research articles were collected from civilian populations, we also included two military articles and two pediatric population articles. The injury mechanisms and populations were not formally extracted. Future research should prioritize stratified analyses by injury mechanism and population subtype to refine prognostic modeling. Lastly, there may be an inherent selection bias stemming from reviewers’ prior beliefs, which can influence the studies included in the manuscript. This was balanced by including authors from diverse medical backgrounds and at different stages of their research careers.

## 5. Conclusions

This review article lays the groundwork for understanding mTBI in the context of poor prognostic factors. Future studies should evaluate firm timeframes for achieving maximal medical benefit in mTBI and the impact of workers’ compensation cases on potential interference with reaching maximal medical benefit.

## Figures and Tables

**Table 1:** Classification of Mild Traumatic Brain Injury. Diagnostic criterion of mild traumatic brain injury as classified by the American Congress of Rehabilitation Medicine and adopted by WHO [12,13]. Although these criteria provide standardized diagnostic thresholds, they do not independently predict long-term functional recovery.

Classification Criterion	Definition for mTBI
Glassgow Coma Score (GCS)	13–15 (measured ≥ 30 min post-injury)
Loss of Consciousness (LOC)	0–30 mins
Post-Traumatic Amnesia Duration	<24 h
CT Imaging	No structural changes

## Data Availability

Not applicable review article.

## Supplementary Materials

The following supporting information can be downloaded at https://www.mdpi.com/article/10.3390/brainsci16030273/s1. File S1. Full Electronic Search Strategy.

## Abbreviations

The following abbreviations are used in this manuscript:

mTBI	Mild Traumatic Brain Injury
CTE	Chronic Traumatic Encephalopathy
PTA	Post-traumatic amnesia
WHO	World Health Organization
WPTAS	Westmead Post-Traumatic Amnesia Scale
GOAT	Galveston Orientation and Amnesia Test
LOC	Loss of consciousness
GCS	Glasgow Coma Scale
CT	Computed Tomography
MRI	Magnetic Resonance Imaging
qEEG	Quantitative EEG
GFAP	Glial Fibrillary Acidic Protein
UCH-L1	Cbiquitin C-terminal Hydrolase
BDNF	Brain-Derived Neurotrophic Factor
BCTT	Buffalo Concussion Treadmill Test
CGRP	Calcitonin Gene-Related Peptide
FDA	Food and Drug Administration
SSRIs	Selective Serotonin Reuptake Inhibitors
CBT	Cognitive Behavioral Therapy
VPT	Vestibular Physical Therapy
PTSD	Post-traumatic Stress Disorder
MDD	Major Depression
APOE	Apolipoprotein E
