# Supplementary material for: Management and Prognosis of Patients with Mild Traumatic Brain Injury: A Narrative Review
Source: Brain Sci. 2026 Feb 28;16(3):273. doi: 10.3390/brainsci16030273 (PMC13023580; doi:10.3390/brainsci16030273)
Supplement: Supplementary file 1 [file brainsci-16-00273-s001.zip › brainsci-4149234-SI.pdf]

brain sciences

Supplementary File S1. Full Electronic Search Strategy

Embase

Search conducted: April 1, 2025 – February 22, 2026

Search string:

('mild traumatic brain injury' OR 'mTBI' OR 'concussion')
AND
('prognosis' OR 'prognostic factors' OR 'recovery')
AND
('diagnosis' OR 'biomarkers' OR 'GFAP' OR 'UCH-L1' OR 'diffusion tensor imaging' OR 'quantitative EEG')
AND
('management' OR 'treatment' OR 'graded exercise' OR 'vestibular rehabilitation' OR 'cognitive behavioral therapy')

Limits:

- Human studies
- English language

PubMed/MEDLINE

Search conducted: April 1, 2025 – February 22, 2026

Search string:

("mild traumatic brain injury" OR "mTBI" OR "concussion")
AND
("prognosis" OR "prognostic factors" OR "recovery")
AND
("diagnosis" OR "diagnostic criteria" OR "biomarkers" OR "GFAP" OR "UCH-L1" OR "diffusion tensor imaging" OR "DTI" OR "quantitative EEG")
AND
("management" OR "treatment" OR "graded exercise" OR "vestibular therapy" OR "cognitive behavioral therapy")

Filters applied:

- Humans
- English language

- Peer-reviewed articles

Google Scholar

Search conducted: April 1, 2025 – February 22, 2026

Search terms:

"mild traumatic brain injury" prognosis management biomarkers

Screening was limited to the first 200 results sorted by relevance.
